# Comparison of the Bedside Head-Impulse Test with the Video Head-Impulse Test in a Clinical Practice Setting: A Prospective Study of 500 Outpatients

**DOI:** 10.3389/fneur.2016.00058

**Published:** 2016-04-20

**Authors:** Chun Wai Yip, Miriam Glaser, Claudia Frenzel, Otmar Bayer, Michael Strupp

**Affiliations:** ^1^Department of Neurology, German Center for Vertigo and Balance Disorders, University Hospital Munich, Munich, Germany; ^2^National Neuroscience Institute, Singapore General Hospital Campus, Singapore

**Keywords:** head-impulse test, sensitivity, positive predictive value, specificity, negative predictive value, vestibulo-ocular reflex

## Abstract

**Objectives:**

The primary aim was to determine the sensitivity, specificity, positive predictive value (PPV), and negative predictive value (NPV) of the bedside head-impulse test (bHIT) using the video HIT (vHIT) as the gold standard for quantifying the function of the vestibulo-ocular reflex (VOR). Secondary aims were to determine the bHIT inter-rater reliability and sensitivity in detecting unilateral and bilateral vestibulopathy.

**Methods:**

In this prospective study, 500 consecutive outpatients presenting to a tertiary neuro-otology clinic with vertigo or dizziness of various vestibular etiologies who did not have any of the pre-defined exclusion criteria were recruited. Bedside HITs were done by three experienced neuro-otology clinicians masked to the diagnosis, and the results were compared with the vHIT. The patients were likewise blinded to the bHIT and vHIT findings. Patients with VOR deficits were identified on the vHIT by referencing to the pre-selected “pathological” gain of <0.7. The data were then analyzed using standard statistical methods.

**Results:**

For the primary outcome (vHIT “pathological” VOR gain <0.7), the three-rater mean bHIT sensitivity = 66.0%, PPV = 44.3%, specificity = 86.2%, and NPV = 93.9%. Shifting the “pathological” threshold from 0.6 to 0.9 caused the bHIT sensitivity to decrease while the PPV increased. Specificity and NPV tended to remain stable. Inter-rater agreement was moderate (Krippendorff’s alpha = 0.54). For unilateral vestibulopathy, overall bHIT sensitivity = 69.6%, reaching 86.67% for severely reduced unilateral gain. For VOR asymmetry <40% and >40%, the bHIT sensitivity = 51.7 and 83%, respectively. For bilateral vestibulopathy, overall bHIT sensitivity = 66.3%, reaching 86.84% for severely reduced bidirectional gains.

**Conclusion:**

For the primary outcome, the bHIT had moderate sensitivity and low PPV. While the study did not elucidate the best choice for vHIT reference, it demonstrated how the bHIT test properties varied with vHIT thresholds: selecting a lower threshold improved the sensitivity but diminished the PPV, while a higher threshold had the opposite effect. The VOR was most likely normal if the bHIT was negative due to its high NPV. The bHIT was moderately sensitive for detecting unilateral and bilateral vestibulopathy overall, but better for certain subgroups.

## Introduction

In 1988, a bedside test for the angular vestibulo-ocular reflex (aVOR) was described: the head thrust or head-impulse test (HIT) (1, 2). The patient’s head was quickly turned to the right or left, and the examiner looked whether the patient’s eyes stayed on the target, or the patient made re-fixation saccades, which would indicate a high-frequency aVOR deficit. This has been assumed to be the clinical standard for the bedside examination of the aVOR. However, it was shown that the bedside HIT (bHIT) can be flawed because it failed to detect “covert saccades” (3). This clinically imperceptible eye movement typically occurred in vestibular-deficient patients who could generate a very early saccade during the first 100 ms of the HIT, and its occurrence seemed to increase with increasing head-turning velocity (4). Furthermore, patients with cerebellar (flocculus) dysfunction tended to have a mild centrally mediated VOR deficit, resulting in bilateral falsely pathological bHIT (5).

The gold standard for measuring the function of the aVOR is the magnetic scleral search coil technique (6, 7). Since this is semi-invasive and only a few centers are able use this method, the video-HIT was developed by different groups to enhance bedside clinical testing (3, 8). Direct comparisons between the magnetic scleral search coil technique and the vHIT showed that the latter was a reliable test (3, 9), which is now being widely used.

In a study on 24 subjects, the bHIT was videotaped and compared with scleral search coil recording (10). Experts and non-expert raters had to view and classify a pre-recorded bHIT. It was found that experts were more conservative when interpreting the bHIT, resulting in a lower sensitivity compared to “non-experts” (63 vs. 72%), but a higher specificity (78 vs. 64%) compared to the latter. In another study on 179 patients, the sensitivity of the bHIT was 35%, and the specificity was 92%, when referenced to the vHIT (11).

The primary objective of this prospective double-blind study was to evaluate the sensitivity, specificity, positive predictive value (PPV), and negative predictive value (NPV) of the bHIT in a large cohort of 500 consecutive outpatients in a real clinic setting, by defining all “pathological” cases as having a vHIT gain <0.7. The secondary objectives were to (i) determine how the bHIT parameters would change when the user-defined “pathological” vHIT cutoff gain was varied from 0.6 to 0.9, since there is no general agreement about what value constitutes a pathological vHIT (although there is normative data for healthy subjects up to 90 years old showing mean horizontal VOR gain to be close to 1 with small SDs) (12, 13); (ii) assess the inter-rater reliability of the bHIT performed by multiple trained raters; and (iii) evaluate the bHIT sensitivity in patients with unilateral vestibulopathy and bilateral vestibulopathy.

## Materials and Methods

### Participants

#### Inclusion Critieria

All patients who attended the vertigo clinic at the Department of Neurology at the University Hospital Munich, Campus Grosshadern, for vertigo, dizziness, or imbalance regardless of etiology, from 1 April 2014 were screened until 500 patients with analyzable data who did not meet any of the exclusion criteria were recruited. Their diagnoses are summarized in Table 1.

**Table 1:** **Demographics and diagnoses of study participants**.

	No. of subjects	Percentage
**Study subjects**	500	
Males	293	58.60
Age range (in years)	29–96	
Females	207	41.40
Age range (in years)	23–92	
**Disease classification**		
Menière’s disease	106	21.20
BPPV	72	14.40
Functional dizziness	65	13.00
Unilateral vestibulopathy	52	10.40
Vestibular migraine	36	7.20
Central nystagmus	32	6.40
Cerebellar ataxia syndrome	26	5.20
Bilateral peripheral vestibulopathy	23	4.60
Vestibular paroxysmia	20	4.00
Central gait disorder	15	3.00
Unknown etiology	27	5.40
Brainstem lesion	11	2.20
Post-traumatic dizziness	7	1.40
Vestibular schwannoma	7	1.40
Episodic ataxia type 2	1	0.20

#### Exclusion Criteria

Patients in whom the bHIT or vHIT could not be performed, mainly due to restricted movement of the head (degenerative cervical spine), disordered right eye motility (oculomotor palsy), congenital nystagmus, or poor vision in the right eye. This was because the infra-red video camera was built into the right side of the goggle frame used to measure the VOR and only tracked the movement of the right eye.

### Study Protocol

The study was conducted prospectively, and both the bHIT and vHIT were performed as part of the existing standard patient consultation. Three experienced vertigo clinic staff clinician raters A, B, and C (who were also the coauthors of the paper) were designated to perform the bHIT for the entire study duration. The examiners and patients were masked to the diagnosis and presence or absence of vestibular dysfunction when they first performed the bHIT. A brief explanation was first given to each patient about the bHIT and vHIT procedure. Next, a quick eye movement examination was performed to ensure the patient could make saccades and at least count fingers at 1 m. Without asking for further history, the bHIT was first performed. At least two out of the three designated raters performed the bHIT in the clinic during the same consultation, and separately documented their responses, after which the vHIT was performed by rater A. Subsequently, the usual full neuro-otological assessment and consultation was completed by rater A, with the patient being given a final diagnosis and treatment.

This study was carried out in accordance with the recommendations of the local hospital ethics committee, at the University of Munich, with patients’ informed verbal consent but with a waiver of formal written informed consent.

#### Bedside Head-Impulse Test

The subjects were seated upright facing the examiner on an examining couch with their eyes at the same level as the examiner. The patients were informed about the procedure and instructed to look at the examiner’s nose at all times during the bHIT. The examiner held the patient’s head firmly with both hands on the temporal–parietal regions. Starting from the straight ahead position, the patient’s head was rapidly turned (HIT) either to the right or to the left by 15–20° in a completely random manner so as to prevent the patient from predicting the direction of the test. The patient’s eyes were observed at the end of the head turn for their final position of rest and the occurrence of a re-fixation saccade, which indicated a VOR deficit. The examiner performed the bHIT two to three times in each direction until a consistent response was obtained. The bHIT was deemed abnormal if a re-fixation saccade was detected by visual inspection.

#### Video-Oculography

To measure the subjects’ eye movements, we used the portable vHIT device (GN Otometrics, ICS Impulse, USA). This consisted of a pair of light-weight video goggles containing an inertial accelerometer, an infra-red video camera on the right side of the frame, and a laser mount. The goggles were secured tightly with adjustable straps to prevent slippage during the vHIT. During the calibration process, patients were instructed to make saccades to a laser visual target projected from the laser beam mounted on the goggles, which alternated between the right and left side. The angular VOR response during the vHIT was recorded by the infra-red camera (at 250 Hz) and displayed on a laptop in real-time. The OtosuiteV^®^ software automatically calculated the VOR gain and displayed the eye and head velocity curves graphically (option of 2D or 3D view) on the screen.

#### Video Head-Impulse Test

Patients sat 1 m in front of a wall on which a small brightly colored target was affixed at eye level. After eye movement calibration of the vHIT system, vHITs were performed by the examiner who stood behind the seated patient and held both sides of the patient’s head in the temporal–parietal region. The head-impulse test was done randomly toward either direction (leftward and rightward head turns), while the patient was instructed to fixate on the wall target. The examiner performed up to 10 head impulses until the software algorithm captured and processed 7 correctly done head impulses in each direction. No extra appliances were used to prevent goggle slippage (e.g., band aids or dental paste over the nose, bite supports) apart from securing the goggle straps tightly and keeping the hands off the straps during the HIT. The software analysis algorithm calculated the gain based on the ratio of the area under the desaccaded eye velocity curve against the area under the head velocity curve, from the time the head was turned until it came to a stop (i.e., when the head velocity profile curve crossed the baseline again), and the average of seven gains was displayed. The velocity profiles for the head and eye movements, together with the presence of re-fixation saccade eye movements, were also displayed on screen.

### Statistical Analysis

The bHIT result obtained by each examiner was compared with the corresponding vHIT for each direction of the VOR. Hence, each patient had two bHITs (right and leftward) done by each rater and two vHITs for comparison. Each patient’s VOR gain in either direction of head impulse was classified as either “pathological” or “normal” with reference to the pre-selected vHIT gain setting. For each individual rater, all corresponding pairs of bHIT and vHIT (for each direction) were grouped together and sorted into a 2 × 2 table to obtain values for true positive (TP) cases, true negative (TN) cases, false positive (FP) cases, and false negative (FN) cases. (For the primary outcome analysis, cases were classified as “pathological” if their vHIT gain was <0.7.) To calculate the three-rater mean sensitivity, mean specificity, mean PPV, and mean NPV, the data from the three raters were pooled together to obtain the total TP cases, total TN cases, total FP cases and total FN cases, and re-analyzed again as above.

The individual rater’s bHIT sensitivity, specificity, PPV, and NPV were re-calculated for different vHIT-defined “pathological” gains from 0.6 to 0.9 to show how the performance parameters varied with changing the gain threshold definition.

The inter-rater reliability for three raters was estimated by means of Krippendorff’s alpha Kα (on SPSS version 18, with an additional macro downloaded free from the author A. F. Hayes’ website) to assess the consistency of the raters’ clinical observation when performing the bHIT.

A subset of patients (*n* = 43) with unilateral vestibulopathy (of various etiologies) defined in this study with unilateral vHIT gain <0.7 was selected to evaluate the overall bHIT sensitivity for this condition and to test how the bHIT sensitivity varied with the VOR asymmetry (calculated as [vHIT_higher value_ − vHIT_lower value_]/vHIT_higher value_, and expressed as a percentage) and VOR gain of the affected labyrinth. Similarly, another subset of patients with bilateral vestibulopathy (defined in this study as bilateral vHIT gains <0.7) of any cause (*n* = 38) were analyzed for the overall bHIT sensitivity and how the bHIT sensitivity varied with the different degrees of bilateral hypofunction and VOR asymmetry.

## Results

**Figure 1:**
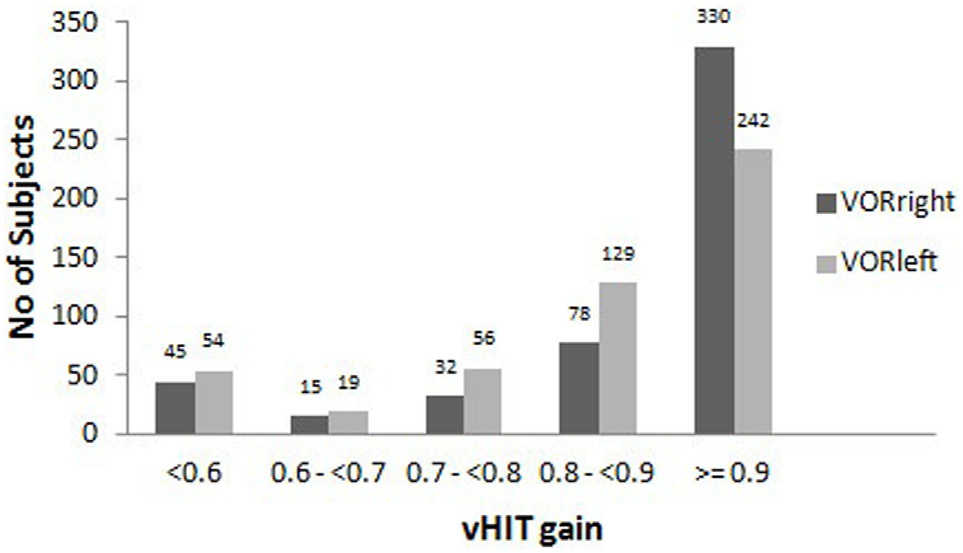
**The distribution of vHIT gains in the study group**.

We screened 510 patients from 1 April 2014 to 28 February 2015 and tested 500 patients who fulfilled the inclusion/exclusion criteria. A total of 1000 vHITs (500 patients each with rightward and leftward vHIT, Figure 1) and 2546 bHITS were performed (not every patient was examined by all 3 raters).

In the primary outcome, for a vHIT “pathological” cutoff gain <0.7, the bHIT sensitivity obtained for raters A, B, and C was 68.1, 61.5, and 67.5%, respectively (mean bHIT sensitivity = 66.0%), specificity was 90.9, 90.1, and 76.7%, respectively (mean specificity = 86.2%), PPV was 55.2, 50.4, and 33.1%, respectively (mean PPV = 44.3%), and NPV was 94.6, 93.5, and 93.3%, respectively (mean NPV = 93.9%).

**Figure 2:**
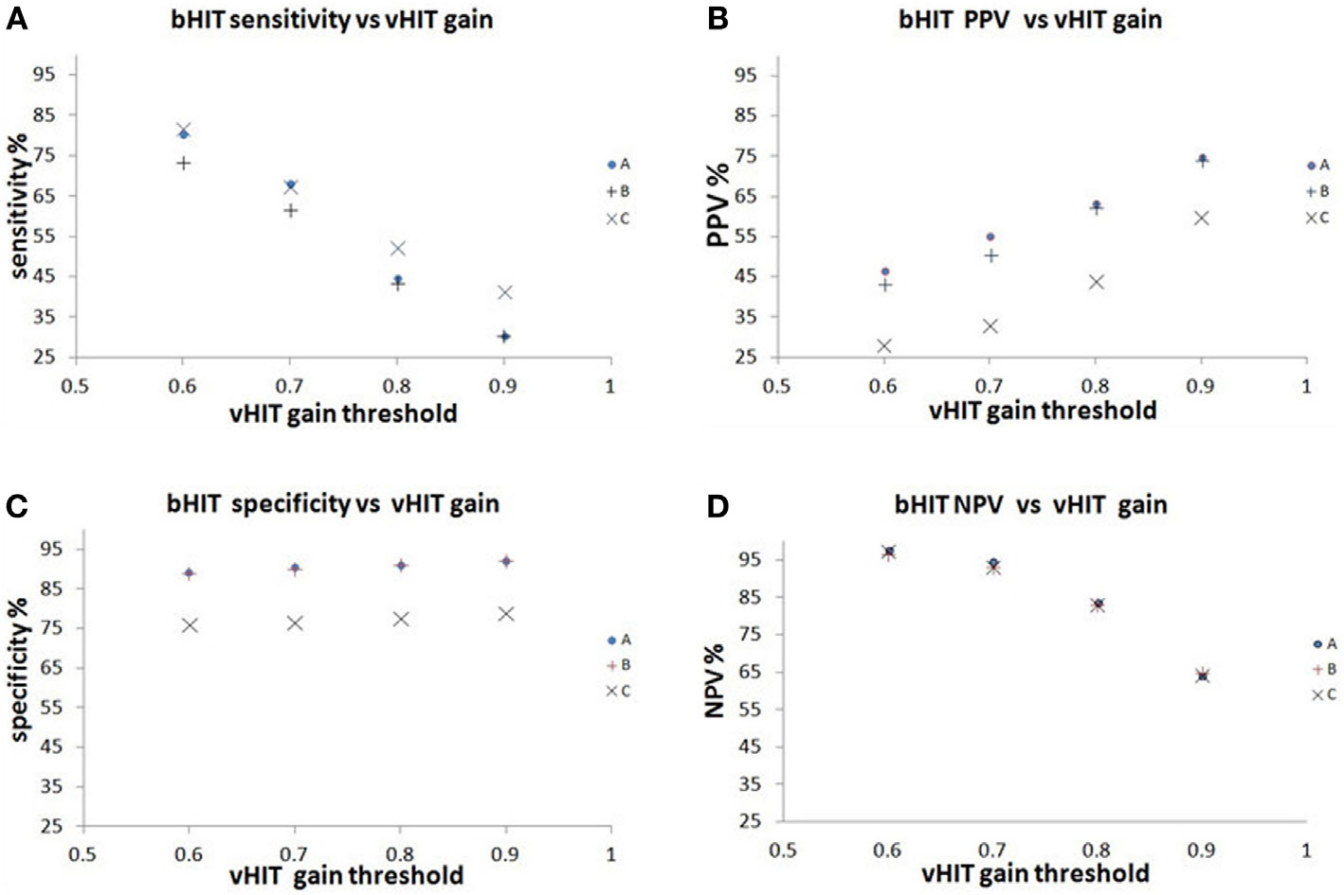
**The relationship of the bHIT sensitivity, PPV, specificity and NPV to different vHIT pathological gain thresholds from 0.6 to 0.9**. **(A)** The bHIT sensitivity decreased with increasing vHIT gain setting from 0.6 to 0.9. **(B)** The bHIT PPV increased with increasing vHIT gain setting from 0.6 to 0.9. **(C)** The bHIT specificity remains above 76% for vHIT gains 0.6 to 0.9. **(D)** The NPV remained above 80% until vHIT gain setting was >0.8.

The bHIT sensitivity, specificity, PPV, and NPV for a range of vHIT gain thresholds from 0.6 to 0.9 are tabulated in Table 2 and graphically shown in Figures 2A–D. In general, the bHIT sensitivity tended to decrease with increasing vHIT “pathological” gain setting, while the PPV tended to increase. The NPV remained >80% until the vHIT gain setting was >0.8, but the specificity remained >76% across all gain settings applied.

**Table 2:** **Individual rater TP, FP, FN, TN, and sensitivity, specificity, PPV, and NPV tabulated for each increasing setting of the “pathological” vHIT gain from 0.6 to 0.9**.

	TP	FN	FP	TN	Sensitivity (%)	Specificity (%)	PPV (%)	NPV (%)
**vHIT gain <0.6**
A	81	20	93	806	80.2	89.66	46.55	97.58
B	55	20	72	595	73.33	89.21	43.31	96.75
C	67	15	172	550	81.71	76.18	28.03	97.35
**vHIT gain <0.7**
A	96	45	78	781	68.09	90.92	55.17	94.55
B	64	40	63	575	61.54	90.13	50.39	93.50
C	79	38	160	527	67.52	76.71	33.05	93.27
**vHIT gain <0.8**
A	110	136	64	690	44.72	91.51	63.22	83.54
B	79	103	48	512	43.41	91.43	62.20	83.25
C	105	95	134	470	52.5	77.81	43.93	83.19
**vHIT gain <0.9**
A	130	297	44	529	30.44	92.32	74.71	64.04
B	94	216	33	399	30.32	92.36	74.02	64.88
C	143	202	96	363	41.45	79.08	59.83	64.25

The inter-rater reliability between the three raters was moderate (Krippendorff’s alpha, Kα = 0.54), reflecting the differences in opinion among multiple raters when interpreting the bHIT. Further sub-analysis showed that the inter-rater agreement between A and B (Kα = 0.74) was good, but it was poor between B and C (Kα = 0.46), and A and C (Kα = 0.43). This was due to rater C diagnosing more false positives than the others.

**Figure 3:**
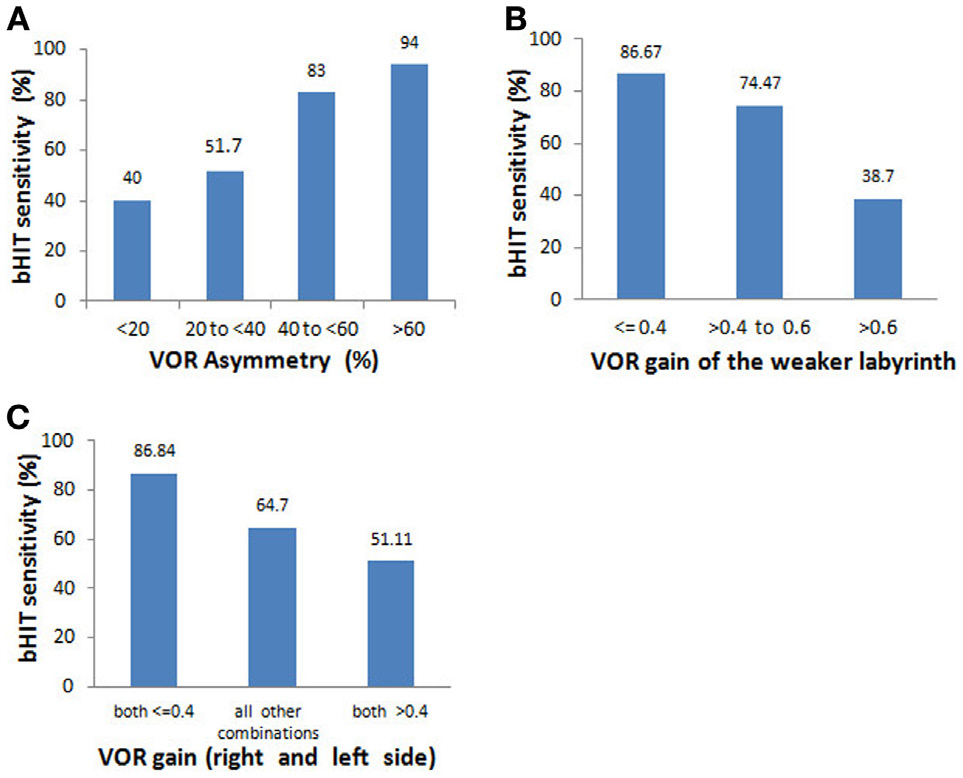
**Relationship of bHIT sensitivity to the VOR asymmetry and absolute VOR gains in unilateral vestibulopathy and bilateral vestibulopathy**. Subgroup analysis of patients with unilateral vestibulopathy (*n* = 43) and bilateral vestibulopathy (*n* = 38). **(A)** The bHIT sensitivity increased with increasing VOR asymmetry between the 2 labyrinths. **(B)** The bHIT sensitivity increased with increasing degrees of unilateral hypofunction. **(C)** When both labyrinths had severe VOR gain reductions, the bHIT had the highest sensitivity. The bHIT sensitivity was most dependent on the VOR asymmetry in the group with dissimilar gains (“all other combinations”).

The overall bHIT sensitivity in patients with unilateral vestibulopathy (*n* = 43) was 69.6%, but tended to improve with increasing VOR asymmetry: for a VOR asymmetry of 1 to <20%, the bHIT sensitivity = 40%; for a VOR asymmetry of 20 to <40%, the bHIT sensitivity = 51.7%; and for a VOR asymmetry of 40% and above, the bHIT sensitivity = 83% or more (Figure 3A). Also, when the weaker labyrinth’s VOR gain was (i) ≤0.4, the bHIT sensitivity = 86.67%, (ii) between 0.4 to 0.6, the bHIT sensitivity = 76.47%, and (iii) more than 0.6, the bHIT sensitivity = 38.7% (Figure 3B). These three groups had median VOR asymmetries of 64.8, 41.23, and 22.6%, respectively, which were significantly different (Mann–Whitney *U* test *p* = 0.001). The VOR asymmetry was inversely related to the VOR gain of the weaker labyrinth and both showed a consistent effect on the inherent ability of the bHIT to detect abnormality. The overall sensitivity for bilateral vestibulopathy of all causes (*n* = 38) was 66.3%. The bilateral vestibulopathy cases were further subcategorized into three groups: (i) both VOR gains ≤0.4, (ii) both VOR gains more than 0.4, and (iii) all other VOR gain combinations (e.g., 0.1 and 0.6). The bHIT sensitivities were 86.84, 51.11, and 64.7%, respectively for each group (Figure 3C). The median VOR asymmetry for each group was 6.06, 12.3, and 20.51%, respectively, but this was significantly different only between groups (i) and (iii) (Mann–Whitney *U* test *p* = 0.017).

## Discussion

The major findings of this study are summarized as follows: first, the bHIT sensitivity for detecting vestibular hypofunction was moderate (for “pathological” vHIT cutoff gain <0.7) even in the hands of experienced neuro-otology clinicians and confirmed the results of a previous study which compared the bHIT with sclera search coil recordings (10). The “pathological” vHIT cutoff was selected from within the range of values used by other published studies that have defined “pathological” gains between 0.6 and 0.86 (3, 4, 11). Second, there were enough bHIT false positives that caused the mean PPV to be low (44.3%), but the PPV tended to improve with increasing vHIT thresholds used for defining “pathological” VOR. Third, the bHIT consistently produced a high mean NPV across the range of vHIT thresholds and decreased somewhat only after 0.8. (The low prevalence of vestibular hypofunction in the study would not completely explain why the bHIT NPV remained robust enough even at the higher thresholds where the number of FN also increased.) Nevertheless, a negative bHIT would mostly indicate that there was no significant VOR deficit. Fourth, the bHIT had a high mean specificity, which remained high across all vHIT cutoff values from 0.6 to 0.9. Fifth, the inter-rater agreement is moderate (mainly due to the effect of rater C, who had good bHIT specificity but poor PPV) and suggested that interpreting the bHIT visually was subjective and not as straightforward as commonly thought. Although rater C had a much lower PPV (due to a higher rate of FP), that data were included in the analysis because this situation reflected clinical practice, since the bHIT is a test where subjectivity is inevitable. Finally, an abnormal VOR was easier to detect if the deficit was unilateral with low gain and large asymmetry between the two labyrinths, or there was bilateral severe vestibular hypofunction.

Despite its potential myriad shortcomings, the bHIT has been regarded as a clinically useful test and the only bedside test for the examination of the high-frequency VOR, in order to detect a unilateral peripheral vestibular deficit (e.g., acute unilateral vestibulopathy/vestibular neuritis) or a bilateral peripheral vestibular deficit (e.g., bilateral gentamicin-induced vestibulopathy) since its first description in 1988. It was also used to differentiate between a peripheral and central vestibular lesion in patients with acute vertigo (2, 14, 15). Only a few other studies have tried to compare the bHIT with more objective measures of VOR gain. Therefore, our study is unique and fills in this practice gap, by analyzing data from a very large cohort and using multiple raters.

The fundamental limitations in interpreting the bHIT are largely caused by the patient’s level of attentiveness, the examiner’s skill in performing the HIT (e.g., adequate head acceleration, small amplitude, randomly directed), and ability to observe re-fixation saccades, as well as the presence of covert saccades, which are impossible to detect visually (16). In this regard, the moderate bHIT sensitivity of our study was not surprising. *Post hoc* analysis of our FN cases (for the primary outcome) revealed that their median gains were significantly higher than the TP cases (0.6 vs. 0.42, Mann–Whitney *U* test *p* = 0.001), which would have resulted in smaller eye corrections and hence harder to detect. The low PPV, on the other hand, was unexpected. This could be due to the predominance of conditions, which would have yielded normal bHITs (e.g., BPPV, vestibular migraine, early stage Menière’s disease, psychogenic dizziness, etc.) in our study cohort, resulting in a low pre-test prevalence of cases with hypofunction. However, by shifting the definition of the “pathological” vHIT gain threshold, the study prevalence of abnormal VOR could be altered. For example, shifting the vHIT cutoff toward 0.9 would “enrich” the cohort with “abnormal” VOR (TP) and improve the PPV. Similarly, selecting a low vHIT threshold would in effect further reduce the cases with “abnormal” VOR, making a positive bHIT test more likely to be detecting false positives instead (leading to poorer PPV).

The practical usefulness of the bHIT was demonstrated particularly by evaluating the bHIT sensitivity for the subgroups with unilateral and bilateral vestibulopathy. The sub-analysis of patients with vHIT-confirmed unilateral vestibulopathy showed that the bHIT sensitivity was 69.6% overall. The bHIT sensitivity was found to be driven by 2 (inversely) related measures – VOR asymmetry and absolute VOR gain. The bHIT sensitivity was 51.7% for cases with a VOR asymmetry of up to 40% and bHIT sensitivity reached 83% or more for VOR asymmetry of more than 40%. Expectedly, the bHIT was superior (sensitivity = 86.84%) at detecting unilateral vestibulopathy cases with very diminished unilateral gain (≤0.4), while it only detected about half the cases with mild unilateral vestibulopathy. For bilateral vestibulopathy, the overall bHIT sensitivity was 66.3%, and the bHIT sensitivity was highest in the cases with bilateral severe hypofunction although the VOR asymmetry was low. The data suggested that the bHIT may be particularly useful in certain situations for different reasons: for bilateral vestibulopathy, the bHIT was most sensitive at detecting bilateral severe vestibular hypofunction compared to bilateral mild vestibular hypofunction, despite the small VOR asymmetries in both subgroups (i.e., the actual VOR gain was important). The effect of a larger VOR asymmetry seemed to contribute to better detection of bilateral vestibulopathy when the deficits were sufficiently different between the two labyrinths (i.e., “all other combinations”). For unilateral vestibulopathy, the VOR asymmetry between the normal and weaker labyrinth was the predominant factor (although the study did not specifically examine re-fixation saccades for further correlation with the above findings).

The study had some limitations. First, the majority of patients could be expected to have normal bHITs because most of the presenting conditions did not cause VOR deficits (i.e., low study prevalence). But this was considered typical of neuro-otology practice.

Second, only experts performed the bHIT for this study. In practice, the bHIT performance characteristics might be worse if performed by less experienced clinicians. Therefore, a future study could include non-experts to perform the testing.

Third, although there were small gain differences between the normal right and leftward head impulses while recording unilaterally from the right eye, the same cutoff gain was applied for both directions of the vHIT analysis for practical reasons. A separate *post hoc* analysis of a subset of our “controls” with normal VOR consisting of BPPV and psychogenic dizziness (*n* = 78, outliers excluded), showed that the rightward (right eye adducting) vHIT gain (mean = 0.965, SD = 0.09, 95% CI 0.95, 0.99) was marginally higher than the leftward (right eye abducting) vHIT gain (mean = 0.904, SD = 0.084, 95% CI 0.89, 0.92) with a mean 6.3% directional asymmetry (both data sets were normally distributed). However, even adjusting for this finding would not have affected the overall behavior trend of the bHIT that we have demonstrated.

The strengths of the study were the largest sample size reported thus far for this kind of study, use of multiple raters to show how the bHIT varied between clinicians, testing conducted in a clinical practice setting with few exclusion criteria, thereby increasing its generalizability.

In conclusion, this prospective double-blinded study performed in a clinical practice setting demonstrates that the bHIT has, at most, moderate sensitivity for detecting VOR deficits even by experienced clinicians, but is a good test for identifying most normal cases. This study was not meant to search for the optimal “pathological” vHIT reference. We have instead demonstrated that the test properties of the bHIT were dependent on the *a priori* “pathological” vHIT threshold gain selected. So, in reality the sensitivity could be lower while the PPV could be better than expected if a higher “pathological” vHIT threshold was used. This partly accounts for why different studies so far have reported different test sensitivities of the bHIT. Our study found that the bHIT had surprisingly good sensitivity in two situations: bilateral severe vestibular hypofunction and unilateral severe hypofunction. However, as a whole, the bHIT sensitivity for unilateral vestibulopathy and bilateral vestibulopathy was moderate. Mild cases of unilateral and bilateral vestibulopathy with small VOR asymmetry would still be missed by the bHIT about half of the time. For all the above reasons, in order to improve on the bHIT test deficiencies, we recommend that vHIT should be used routinely to complement the bHIT.

## Author Contributions

CY: contributed to data acquisition, statistical analysis and interpretation of results, drafting of the manuscript, revised the manuscript and approved the final manuscript. MG and CF: contributed to data acquisition, revised and approved the final manuscript. OB: contributed to the statistical analysis, critically reviewed the manuscript and approved the final manuscript. MS: conceptualized and designed the study, contributed to data acquisition, critically reviewed and approved the final manuscript. All authors are agreeable to be accountable for the content of the work, integrity and accuracy of the data.

## Conflict of Interest Statement

MS is Joint Chief Editor of the Journal of Neurology, Editor in Chief of Frontiers of Neuro-otology and Section Editor of F1000. He has received speaker’s honoraria from Abbott, Actelion, Biogen, Eisai, GSK, Henning Pharma, Interacoustics, MSD, Otometrics, Pierre-Fabre, TEVA, and UCB. The remaining authors declare that the research was conducted in the absence of any commercial or financial relationships that could be construed as a potential conflict of interest.

## References

[1] Halmagyi GM Curthoys IS A clinical sign of canal paresis. Arch Neurol (1988) 45:737–9. 10.1001/archneur.1988.00520310043015 3390028

[2] Cnyrim CD Newman-Toker D Karch C Brandt T Strupp M. Bedside differentiation of vestibular neuritis from central “vestibular pseudoneuritis”. J Neurol Neurosurg Psychiatry (2008) 79:458–60. 10.1136/jnnp.2007.123596 18344397

[3] MacDougall HG Weber KP McGarvie LA Halmagyi GM Curthoys IS. The video head impulse test: diagnostic accuracy in peripheral vestibulopathy. Neurology (2009) 73:1134–41. 10.1212/WNL.0b013e3181bacf85 19805730 PMC2890997

[4] Weber KP Aw ST Todd MJ McGarvie LA Curthoys IS Halmagyi GM. Head impulse test in unilateral vestibular loss: vestibulo-ocular reflex and catch-up saccades. Neurology (2008) 70:454–63. 10.1212/01.wnl.0000299117.48935.2e 18250290

[5] Kremmyda O Kirchner H Glasauer S Brandt T Jahn K Strupp M. False-positive head-impulse test in cerebellar ataxia. Front Neurol (2012) 3:162. 10.3389/fneur.2012.00162 23162531 PMC3495261

[6] Robinson DA A method of measuring eye movement using a scleral search coil in a magnetic field. IEEE Trans Biomed Eng (1963) 10:137–45. 14121113 10.1109/tbmel.1963.4322822

[7] Imai T Sekine K Hattori K Takeda N Koizuka I Nakamae K Comparing the accuracy of video-oculography and the scleral search coil system in human eye movement analysis. Auris Nasus Larynx (2005) 32:3–9. 10.1016/j.anl.2004.11.009 15882818

[8] Bartl K Lehnen N Kohlbecher S Schneider E. Head impulse testing using video-oculography. Ann N Y Acad Sci (2009) 1164:331–3. 10.1111/j.1749-6632.2009.03850.x 19645921

[9] Agrawal Y Schubert MC Migliaccio AA Zee DS Schneider E Lehnen N Evaluation of quantitative head impulse testing using search coils versus video-oculography in older individuals. Otol Neurotol (2014) 35:283–8. 10.1097/MAO.0b013e3182995227 24080977 PMC4532669

[10] Jorns-Haderli M Straumann D Palla A. Accuracy of the bedside head impulse test in detecting vestibular hypofunction. J Neurol Neurosurg Psychiatry (2007) 78:1113–8. 10.1136/jnnp.2006.109512 17220287 PMC2117540

[11] Perez-Fernandez N Gallegos-Constantino V Barona-Lleo L Manrique-Huarte R. Clinical and video-assisted examination of the vestibulo-ocular reflex: a comparative study. Acta Otorrinolaringol Esp (2012) 63:429–35. 10.1016/j.otorri.2012.04.010 22789453

[12] Matino-Soler E Esteller-More E Martin-Sanchez JC Martinez-Sanchez JM Perez-Fernandez M Normative data on angular vestibulo-ocular responses in the yaw axis measured using the video head impulse test. Otol Neurotol (2014) 36:466–71. 10.1097/MAO.0000000000000661 25473958

[13] McGarvie LA MacDougall HG Halmagyi GM Burgess AM Weber KP Curthoys IS. The video head impulse test (vHIT) of semicircular canal function – age-dependent normative values of VOR gain in healthy subjects. Front Neurol (2015) 6:154. 10.3389/fneur.2015.00154 26217301 PMC4495346

[14] Kattah JC Talkad AV Wang DZ Hsieh YH Newman-Toker DE. HINTS to diagnose stroke in the acute vestibular syndrome: three-step bedside oculomotor examination more sensitive than early MRI diffusion-weighted imaging. Stroke (2009) 40:3504–10. 10.1161/STROKEAHA.109.551234 19762709 PMC4593511

[15] Newman-Toker DE Kattah JC Alvernia JE Wang DZ. Normal head impulse test differentiates acute cerebellar strokes from vestibular neuritis. Neurology (2008) 70:2378–85. 10.1212/01.wnl.0000314685.01433.0d 18541870

[16] Blodow A Pannasch S Walther LE. Detection of isolated covert saccades with the video head impulse test in peripheral vestibular disorders. Auris Nasus Larynx (2013) 40:348–51. 10.1016/j.anl.2012.11.002 23245835

